# Comparative Analysis of Akerma and Bouchouk Guergour Olive Oils Varieties: Physicochemical Analysis, Quality, and Bioactivity Evaluation

**DOI:** 10.1002/cbdv.202502264

**Published:** 2026-03-02

**Authors:** Samir Sahli, Nabila Souilah, Hamdi Bendif, Amar Benmahammed, Saliha Dahamna, Ramazan Erenler, Walid Elfalleh, İbrahim Demirtaş, Fehmi Boufahja, Stefania Garzoli

**Affiliations:** ^1^ Laboratory For the Optimization of Agricultural Production in the Subhumid Zone, Faculty of Sciences, Department of Agronomic Sciences University August 20 Skikda Algeria; ^2^ Biology Department, College of Science Imam Mohammad Ibn Saud Islamic University (IMSIU) Riyadh Saudi Arabia; ^3^ Laboratory For the Valorization of Natural Biological Resources, Faculty of Natural and Life Sciences, Department of Plant Biology and Physiology Ferhat Abbas University Sétif Algeria; ^4^ Laboratory of Phytotherapy Applied to Chronic Diseases, Faculty of Natural and Life Sciences, Department of Animal Biology and Physiology Ferhat Abbas University Sétif Algeria; ^5^ Research Laboratories Application and Research Center (ALUM) Iğdır University Iğdır Turkey; ^6^ Department of Chemistry and Technologies of Drug Sapienza University Rome Italy

**Keywords:** Akerma, anti‐inflammatory test, Bouchouk Guergour, GC‐FID, GC‐MS, healing wounds, olive oil

## Abstract

The current study investigated the dynamic of the ripening process, oil yield, physicochemical properties, and wound healing activity of two olive varieties, namely Akerma and Bouchouk Guergour. The ripening index revealed that Akerma matured at a slower rate compared to the Bouchouk Guergour variety. The oil yield peaked at 12.71% for the Akerma variety at the spotted stage and 19.56% for Bouchouk Guergour at the black stage, suggesting earlier harvesting time in short‐growing seasons for the former oil tree. Physicochemical analyses showed compliance with International Olive Council standards, with Akerma exhibiting lower acidity compared to Bouchouk Guergour variety. The peroxide value increased with ripening, indicating higher oxidative susceptibility in mature oils. Gas chromatography‐mass spectrometry (GC‐MS) analysis revealed a high content of monounsaturated fatty acids (MUFAs), particularly oleic acid. Notably, in vivo, evaluations demonstrated significant wound healing effects, with both oils achieving complete recovery by Day 28, particularly in their black stages, and showcasing anti‐inflammatory properties. These findings underscore the importance of varietal selection and optimal harvest timing to enhance oil quality, yield, and therapeutic potential. This research provides valuable insights for olive growers, facilitating the maximization of production and promoting health benefits associated with high‐quality olive oils.

## Graphical abstract

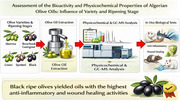

## 1 Introduction

Olive oil, which is derived from the fruits of the olive tree (*Olea europaea* L.), plays a key role in the Mediterranean diet and cuisine and is highly appreciated due to its culinary applications and numerous health benefits. The global market for olive oil has witnessed significant growth in recent years, driven by increasing consumer awareness of the nutritional advantages associated with its consumption. Particularly, this oil comprises a high content of monounsaturated fatty acids (MUFAs) and bioactive compounds, such as phenols and tocopherols [1]. These compounds have therapeutic properties, such as antioxidant activity, anti‐inflammatory effects, and cardiovascular protection [2].

The quality and characteristics of olive oil are influenced by several factors, including the ripening stage, geographic origin, and extraction methods. The olive oil quality is usually measured via well‐established parameters, such as free acidity, peroxide value, and iodine value. These parameters also measure the stability against oxidation and the degree of unsaturation, which in turn need to comply with the standards set by the International Olive Council [3]. Furthermore, the fatty acid composition analyses are very helpful in identifying the sensory properties and health benefits of olive oil.

The study of oil aromas with solid‐phase micro‐extraction (SPME) and gas chromatography‐mass spectrometry (GC‐MS) provides additional valuable information about the volatile compounds that are responsible for the unique aroma flavor of the oil [4]. These compounds contribute to the overall flavor and quality of the oil, making their analysis essential for both producers and consumers.

Beyond sensory properties, olive oil exhibits a variety of biological activities that have garnered significant interest in recent years. Research has demonstrated the anti‐inflammatory properties of olive oil, indicating that its bioactive compounds may modulate the inflammatory pathways [5]. Additionally, the wound‐healing activity of olive oil was also investigated, revealing an efficient compound that enhances the repair and regeneration properties of damaged human tissues [6]. Moreover, recent studies highlight the active role played by olive oil in promoting skin health, with far‐reaching practical applicability in dermatology [7].

Given the diverse geographical and climatic landscape, Algeria is an important producer of olive in the Mediterranean region. Several varieties of olive, such as Chemlal, Akerma, and Bouchouk Guergour, hold significant economic and cultural importance, contributing to the sustainability of local economies and the enhancement of culinary heritage. The olive trees are primarily cultivated in northern Algeria, where local farmers have employed traditional cultivation techniques since historic times. However, the olive oil sector in Algeria faces severe challenges, such as limited investment in modern extraction technologies, with an undesirable impact on the quality of production [8]. This current study aimed to evaluate the quality and bioactive properties of the oil extracted from two different varieties (i.e., Akerma and Bouchouk Guergour) of olive trees, at various ripening stages. Key parameters, such as biochemical analysis, fatty acid composition analysis, aroma analysis using SPME and GC‐MS, fatty acid profiling using gas chromatography‐flame ionization detection (GC‐FID), as well as the evaluation of anti‐inflammatory and wound healing activities of these oils in vivo, were assessed.

## 2 Results and Discussion

Olive oils extracted from two *O. europaea* varieties, namely, Akerma and Bouchouk Guergour, were used at three distinct ripening stages: green, spotted, and black. Several key physicochemical parameters of the oils were analyzed, such as ripening index, oil yield, free acidity, peroxide value, water, and volatile matter content, as well as iodine value. These assessments aimed to better characterize the quality and properties of the oils across different maturity stages (Table 1).

**TABLE 1:** Physicochemical data were analyzed using one‐way analysis of variance (ANOVA) followed by Tukey's multiple comparison post hoc test.

Varieties	Akerma			Bouchouk Guergour		
Stage	Green	Spotted	Black	Green	Spotted	Black
**Ripening index**	1.6 ± 0.08^a^	4.2 ± 0.13^b^	6.9 ± 0.17^c^	1.8 ± 0.09^a^	4.1 ± 0.11^b^	6.2 ± 0.21^c^
**Oil yield (%)**	7.81 ± 0.34^a^	12.71 ± 0.17^b^	11.96 ± 0.1^b^	8.44 ± 0.1^a^	17.47 ± 0.25^c^	19.56 ± 0.22^d^
**Acidity index (%)**	0.481 ± 0.02^b^	0.308 ± 0.02^a^	0.581 ± 0.03^c^	0.522 ± 0.02^b^	0.554 ± 0.02^b^	0.631 ± 0.02^c^
**Peroxide value (meq O_2_/kg)**	3.25 ± 0.2^a^	5.25 ± 0.37^b^	9.5 ± 0.3^c^	9.0 ± 0.25^c^	10.23 ± 0.19^c^ ^d^	12.5 ± 0.41^d^
**Water and volatiles content (%)**	1.06 ± 0.01^b^	0.62 ± 0.01^a^	1.88 ± 0.01^c^	1.34 ± 0.02^b^	2.04 ± 0.02^d^	1.97 ± 0.01^c^
**Iodine value**	47.10 ± 1.07^a^	63.10 ± 0.84^b^	90.96 ± 1.71^c^	32.08 ± 0.6^a^	67.50 ± 0.93^b^	85.09 ± 0.9^c^

*Note*: The diffent letters indicate the statistically significant differences. Differences between means were considered statistically significant at *p* < 0.05.

Statistical analysis revealed significant differences (*p* < 0.05) in all physicochemical parameters of olive oils according to both ripening stage and cultivar. Oil yield and iodine value increased significantly with fruit maturation, whereas peroxide value and free acidity showed a marked increase at the black ripening stage.

### 2.1 Ripening Index

The ripening index is a key indicator of the maturation process of olives, transitioning from green to black stages. In the current study, both olive varieties, namely Akerma and Bouchouk Guergour, respectively, exhibited a clear and progressive increase in ripening index directly with advancing maturity. For Akerma, the ripening index rose from 1.6 ± 0.08 at the green stage to 6.9 ± 0.17 at the black stage. Similarly, the Bouchouk Guergour variety had an increase from 1.8 ± 0.09 to 6.2 ± 0.21 (Table 2). These results indicated a parallel maturation trend of the two varieties, although Bouchouk Guergour reached an advanced phenological stage at lower ripening index values compared to Akerma. This suggested that the former variety of olive trees may have a slightly faster and more efficient ripening process, potentially due to inherent genetic or environmental factors. Such differences in ripening can be considered important for assessing the optimal harvest time to maximize the oil quality and yield.

**TABLE 2:** Changes in ripening index, oil yield, free acidity, peroxide value, water and volatile matter content, and iodine value of Akerma and Bouchouk Guergour olive oils at different ripening stages.

Varieties	Akerma			Bouchouk Guergour		
Stage	Green	Spotted	Black	Green	Spotted	Black
Ripening index	1.60 ± 0.08^a^	4.20 ± 0.13^b^	6.90 ± 0.17^c^	1.80 ± 0.09^a^	4.10 ± 0.11^b^	6.20 ± 0.21^c^
Oil yield (%)	7.81 ± 0.34^a^	12.71 ± 0.17^b^	11.96 ± 0.10^b^	8.44 ± 0.10^a^	17.47 ± 0.25^c^	19.56 ± 0.22^d^
Free acidity (%)	0.48 ± 0.02^a^	0.31 ± 0.02^a^	0.58 ± 0.03^c^	0.52 ± 0.02^b^	0.55 ± 0.02^b^	0.63 ± 0.02^c^
Peroxide value (meq O_2_/kg)	3.25 ± 0.20^a^	5.25 ± 0.37^b^	9.50 ± 0.30^c^	9.00 ± 0.25^c^	10.23 ± 0.19^c^ ^d^	12.5 ± 0.41^d^
Water and volatiles content (%)	1.06 ± 0.01^a^	0.62 ± 0.01^a^	1.88 ± 0.01^c^	1.34 ± 0.02^b^	2.04 ± 0.02^d^	1.97 ± 0.01^c^
Iodine value	47.10 ± 1.07^a^	63.10 ± 0.84^b^	90.96 ± 1.71^c^	32.08 ± 0.60^a^	67.50 ± 0.93^b^	85.09 ± 0.90^c^

*Note*: The different letters indicate statistically significant differences. All values represent mean ± standard deviation (*n* = 3).

The ripening index was similar between the varieties across different ripening stages (*F* value = 0.01, *p* value > 0.05), indicating that despite slight differences in the absolute values of the ripening index between Akerma and Bouchouk Guergour, the overall differences were not statistically significant. The Tukey test further confirmed that no significant pairwise differences existed between ripening stages or varieties. This statistical result reinforces the observation that both varieties follow a similar ripening pattern, albeit with minor phenological variations.

The ripening index of Akerma and Bouchouk Guergour olive varieties revealed important differences in their maturation dynamics. Both varieties showed a progressive increase in ripening index as they matured, with Akerma ranging from 1.6 ± 0.08 at the green stage to 6.9 ± 0.17 at the black stage and Bouchouk Guergour from 1.8 ± 0.09 to 6.2 ± 0.21. Notably, Bouchouk Guergour reached a more advanced ripening stage at a lower index, suggesting a faster maturation process compared to Akerma.

Similar trends in ripening indices were reported for other olive varieties, with genetic and environmental factors playing crucial roles during the maturation process. For example, Dabbou et al. [9] noted similar ripening patterns in the Chemlali variety from Tunisia, which matured more slowly with indices ranging from 2.0 to 5.7, depending on the harvest period and environmental conditions. Such differences in ripening rates are not uncommon and can be influenced by factors such as temperatures, rainfall regime, and soil composition, all of which impact the phenological development.

Additionally, the findings align with research by Ouni et al. [10] on Mediterranean olive varieties, which showed that early‐ripening varieties accumulate oil more quickly, whereas later‐ripening varieties often produce oil with more balanced chemical characteristics, such as lower free acidity and higher oleic acid content. This comparison highlights the strategic advantage of Bouchouk Guergour in regions with shorter growing seasons, where early harvesting is preferable to capture oils with robust flavors and higher phenolic content. In contrast, the Akerma variety, with its slower maturation process, could be more suited for regions where a wider harvest window is beneficial, enabling producers to optimize both yield and oil quality.

The ripening dynamics of olives directly impact both oil yield and chemical composition. Early‐harvested olives typically have higher concentrations of phenolic compounds, which contribute to stronger antioxidant activity and more bitter‐tasting oils. On the other hand, olives harvested at later stages often yield oil with smoother sensory profiles and lower acidity, a pattern noted by Uceda and Hermoso [11] in their study of Andalusian olive cultivars. These observations are reflected in our findings, where Akerma, which matures more slowly, may offer a broader harvest window, allowing producers to adjust the timing to optimize oil yield and quality.

Compared with other studies, the results of this investigation suggest that harvest timing could be optimized based on desired oil characteristics. Bouchouk Guergour, with its faster maturation rate, may be better suited for early harvests that emphasize robust flavors and high phenolic content, while Akerma might be ideal for mid‐to‐late harvests, producing oils with more balanced organoleptic properties.

These differences underscore the importance of understanding the varietal characteristics and their responses to environmental factors, which can help producers fine‐tune cultivation and harvest strategies for optimal olive oil production. Further research into how climatic conditions influence these varieties will offer deeper insights into how ripening patterns can be best leveraged to produce high‐quality olive oils.

### 2.2 Oil Yield

The oil yield varied significantly between the two olive varieties and across different ripening stages. In the case of Akerma, the oil yield peaked at the spotted stage, namely, at 12.71 ± 0.17%. At the green and black stages, Akerma produced lower yields, indicating that the spotted stage represented the optimal ripening phase for oil extraction of this variety. Conversely, Bouchouk Guergour exhibited the maximum oil yield at the black stage, with 19.56 ± 0.22% (Table 2), suggesting that this variety continues to accumulate oil as the ripening progresses, with the black stage being the most productive for oil extraction.

These results suggest that the oil yield is not solely variety‐dependent, but also highly influenced by the ripening stage, with Akerma showing a preference for intermediate stages, whereas Bouchouk Guergour favors the full maturation of olives. The differences between varieties could also be linked to their unique physiological and biochemical processes, influencing the oil accumulation patterns throughout the ripening period. The oil yield (expressed as percentages) did not show significant differences between varieties and across ripening stages (*F* = 1.34, *p* > 0.05), suggesting that further investigations, with larger sample sizes and replicates, are required in further studies.

The current study showed distinct oil yield patterns between the Akerma and Bouchouk Guergour varieties at different ripening stages. Akerma reached its maximum yield (12.71 ± 0.17%) at the spotted stage, while Bouchouk Guergour achieved its peak yield (19.56 ± 0.22%) at the black stage. These variations highlight the importance of understanding ripening dynamics when determining optimal harvest timing for maximizing oil yield. Specifically, Akerma's peak yield at the spotted stage suggests that it could be ideal for regions with shorter growing seasons, where an early harvest may optimize oil yield while maintaining quality. On the other hand, Bouchouk Guergour, which reaches its highest yield at the black stage, may be better suited for regions with longer growing seasons, where a later harvest can be used to capture more oil and enhance phenolic content.

These findings align with previous research on global olive varieties. For example, the Spanish Arbequina variety, which peaks at an intermediate ripening stage, and the Picual variety, similar to Bouchouk Guergour, which maximizes yield at full ripeness, demonstrate how different maturation patterns can influence oil production [12]. Similarly, the Algerian Chemlal variety follows a late‐ripening oil accumulation pattern, comparable to Baccouri et al. [13]. Furthermore, the Kalamata variety from Greece also peaks at full ripeness, reinforcing the trend of later ripening correlating with higher oil yield [14].

### 2.3 Physicochemical Analysis

#### 2.3.1 Free Acidity

The results of the free acidity analysis, expressed as a percentage of oleic acid, revealed information about the quality of the olive oils for both varieties. For the Akerma variety, free acidity increased from 0.308 ± 0.02% at the spotted stage to 0.581 ± 0.03% at the black stage, respectively. In the case of Bouchouk Guergour, the free acidity increased from 0.522 ± 0.02% in the green stage to 0.631 ± 0.02% in the black stage, respectively (Table 2). These acidity levels remained within the quality standards set by the International Olive Council (IOC), confirming that the analyzed oils in the current investigation met the criteria of high‐quality virgin olive oil. The observed trend of increasing acidity directly with fruit maturation may be attributed to the progressive degradation of lipids and higher oxidation rates of fatty acids as the olives advance through the ripening stages. However, the free acidity did not vary between varieties across ripening stages (*F* = 1.7, *p* > 0.05).

The results indicate that free acidity is a crucial parameter in assessing the quality of olive oil. For the Akerma variety, the free acidity ranged from 0.31 ± 0.02% at the spotted stage to 0.58 ± 0.03% at the black stage, whereas the Bouchouk Guergour variety ranged from 0.52 ± 0.02% to 0.63 ± 0.02%, respectively. These findings align with the International Olive Council (IOC) standards, confirming the good quality of the examined oils. The increase in free acidity during maturation can be attributed to lipid degradation and the oxidation of fatty acids, as noted before by Ghanbari et al. [15] and Karam and Ghandour [16]. This phenomenon was corroborated by the work of Goulas and Badji [17], who observed similar trends in other cultivars and suggested that as olives ripen, the hydrolysis of triglycerides occurs, leading to higher free fatty acid concentrations. Furthermore, Ríos and Sánchez [18] highlighted that the composition and quality of olive oil were closely linked to the ripening stage and specific cultivar characteristics. This indicates that the observed variations in acidity may also reflect the unique biochemical composition of each variety, thus influencing the oil yield and quality. Overall, these results contributed to the growing body of literature emphasizing the importance of monitoring free acidity to evaluate olive oil quality.

#### 2.3.2 Peroxide Value

The peroxide value is an important indicator of primary oxidation in olive oils. In the current study, the Akerma variety increased the peroxide value from 3.25 ± 0.2 meq O_2_/kg at the spotted stage to 9.5 ± 0.3 meq O_2_/kg at the black stage. The Bouchouk Guergour variety showed a progressive increase in peroxide value from 9 ± 0.25 meq O_2_/kg at the green stage to 12.5 ± 0.41 meq O_2_/kg at the black stage, respectively (Table 2). These findings suggest that olive oils derived from more mature olives exhibit higher susceptibility to oxidation, corroborating existing literature on the oxidative stability of olive oils. The peroxide values showed significant differences between varieties and across ripening stages (*F* = 6.45, *p* < 0.05). The Tukey test confirmed significant pairwise differences between the green and black stages for both varieties, with higher peroxide values at more advanced ripening stages. These differences statistically suggest that the maturation of olives leads to an increased risk of oxidation, which is critical for determining the optimal harvest time to ensure oil quality.

The observed results indicated that the peroxide value, a key indicator of oxidative deterioration, increased as the olives ripened for both the Akerma and Bouchouk Guergour varieties. Specifically, for Akerma, the peroxide value rose from 3.25 ± 0.2 meq O_2_/kg at the spotted stage to 9.5 ± 0.3 meq O_2_/kg at the black stage. This suggests that oils from more mature olives are more susceptible to oxidative processes, which is consistent with findings by Tsimidou et al. [19], who demonstrated that the maturity of olives directly influences oxidation rates. More mature olives, with their higher concentrations of unsaturated fatty acids, tend to exhibit greater oxidative susceptibility.

Similarly, the Bouchouk Guergour variety showed an increase in peroxide value, from 9 ± 0.25 to 12.5 ± 0.41 meq O_2_/kg, further supporting the concept that ripening increases oxidative instability in olive oil. This finding aligns with Kallithraka et al. [20], who noted that higher peroxide values in olive oils are typically associated with increased fruit ripeness, leading to greater concentrations of free fatty acids and other compounds prone to oxidation.

Additionally, the results are in agreement with the work of Ghanbari et al. [15], who reported that the oxidative stability and overall quality of olive oils decline with fruit maturity due to the promotion of lipid oxidation pathways. Therefore, monitoring peroxide values is essential for assessing the shelf life and quality of olive oils, especially in relation to the ripening stage at harvest.

#### 2.3.3 Water and Volatile Content

The water and volatile content significantly influence the preservation of olive oil. In the current study, the Akerma variety exhibited the lowest water content at the spotted stage, measuring 0.62 ± 0.01%, whereas the Bouchouk Guergour variety had a higher water content of 2.04 ± 0.02% at the same maturation stage (Table 2). These differences in water content between both varieties suggest that environmental factors, such as climatic and soil conditions, may play a crucial role in the oil's composition, potentially affecting its long‐term stability and quality. Water and volatile contents significantly differed between both the varieties and across ripening stages (*F* = 15.32, *p* < 0.01). The Tukey test confirmed significant pairwise differences between varieties at the spotted and black stages, with the Bouchouk Guergour variety consistently showing higher water content compared to Akerma. These results highlight the influence of variety‐specific and environmental factors on water retention in olive oil, which in turn can influence oil preservation capacity as well as exposure to microbial activity and oxidation.

Different superscript letters within the same row indicate statistically significant differences between samples (one‐way analysis of variance (ANOVA) followed by Tukey's multiple comparison post hoc test, *p* < 0.05).

Significant differences (*p* < 0.05) were observed in all quality parameters according to ripening stage and cultivar. Oil yield and iodine value increased significantly with fruit maturation, while peroxide value and free acidity showed higher values at the black ripening stage.

The findings of the current investigation showed distinct trends in water and volatile content between the Akerma and Bouchouk Guergour varieties, which can significantly impact oil preservation. For Akerma, the observed water content of 0.62 ± 0.01% at the spotted stage indicates lower susceptibility to microbial growth and oxidative reactions, thus enhancing its shelf life. Conversely, the higher water content of 2.04 ± 0.02% in Bouchouk Guergour at the spotted stage may render this oil more prone to spoilage and quality degradation due to increased moisture, which can promote the growth of bacteria and mold [21].

Comparing these results with other olive cultivars from different regions enhances our understanding of how environmental conditions influence oil composition. For instance, the study of D'Imperio and Visconti on Coratina olives grown in Italy reported a lower water content of around 0.5% during the optimal harvest period. This lower moisture level contributes to the high oxidative stability of Coratina oil, making it less prone to rancidity than oils with higher water content, such as Bouchouk Guergour. Similarly, previous research by El Riachy et al. [22] on Lebanese olive oils showed that oils from areas with arid climates had water content levels around 0.7%, thus with improved preservation characteristics compared to oils from more humid regions.

These comparisons underscore the importance of considering environmental factors, including soil and climate, whenever assessing the preservation potential of olive oils from different varieties.

#### 2.3.4 Iodine Value

The iodine value is a crucial metric for assessing the degree of unsaturation of fatty acids present in olive oil. In the study, both Akerma and Bouchouk Guergour varieties exhibited significant increases in iodine value directly with the degree of maturation. Specifically, the iodine value of the Akerma variety increased from 47.10 ± 1.07 at the green stage to 90.96 ± 1.71 at the black stage, whereas the Bouchouk Guergour variety showed an increase from 32.08 ± 0.6 at the green stage to 85.09 ± 0.9 at the black stage, respectively (Table 2). These observations indicate that oils extracted from more mature olives possessed a higher proportion of unsaturated fatty acids, which corresponds to an enhanced capacity for iodine absorption as ripening progresses. Iodine values revealed significant differences across ripening stages and varieties (*F* value = 12.34, *p* value < 0.01). Tukey tests identified significant pairwise differences in iodine values between the green and black stages for both varieties, confirming that the increase in unsaturation with ripening was statistically significant.

Understanding these variations is essential for producers aiming to optimize the fatty acid composition of olive oil, as the degree of unsaturation impacts not only nutritional value but also the oil's stability and shelf life.

The observed increase in iodine value with olive maturation indicates a clear relationship between the ripening stage and the unsaturation of fatty acids in olive oils. The significant rise in iodine value for Akerma and Bouchouk Guergour varieties suggests that as olives mature undergo several biochemical changes that enhance the content of unsaturated fatty acids. The Akerma variety, with an iodine value of 90.96 ± 1.71 at the black stage, shows a particularly high degree of unsaturation compared to the Bouchouk Guergour, which reached iodine values of 85.09 ± 0.9.

These findings align with previous studies that emphasized the impact of ripening on the fatty acid composition of olive oil. For example, the research of Kafkaletou et al. [23] reported similar trends to those observed in the current study for the Koroneiki variety, where the iodine value increased significantly from 45.7 in the early stages to 91.2 in fully ripe olives. This enhancement in unsaturated fatty acids was associated with the sensory quality and health benefits of olive oil, as unsaturated fats are known to contribute to heart health and reduce inflammation [24].

Moreover, a comparative analysis with other varieties, such as Picual and Frantoio, reveals that the unsaturation level varies among cultivars. In the study of Gómez‐Alonso et al. [25], the Picual variety demonstrated an iodine value of 87.0 at full ripeness, thus indicating a similar pattern of unsaturation with the current study but with variations that reflect the unique genetic and environmental factors affecting each variety. Overall, the results of the current investigation underscore the importance of olive maturation in enhancing the nutritional profile and quality of olive oil, thus supporting the observation that higher unsaturation levels are beneficial for both culinary applications and health.

### 2.4 Biochemical Analysis

#### 2.4.1 Study of Oil Aromas by SPME and GC‐MS

**FIGURE 1:**
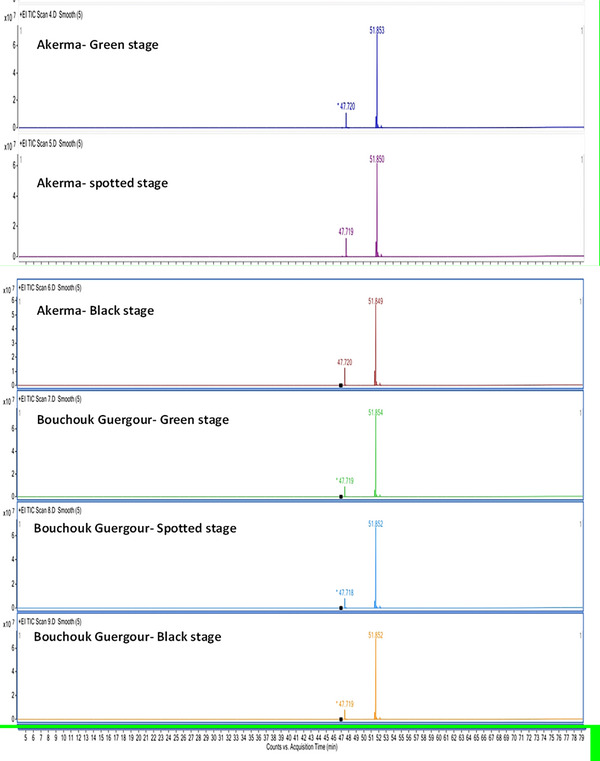
GC‐MS chromatograms illustrating the fatty acid profiles of olive oils from Akerma and Bouchouk Guergour varieties at different ripening stages (green, spotted, and black).

The GC‐MS analysis of the fatty acid composition in olive oils from the Akerma and Bouchouk Guergour varieties revealed notable differences across the maturation stages (Table 3, Figure 1). For Akerma, the oleic acid methyl ester was the dominant compound, comprising between 68.96% and 70.81% across the green, spotted, and black stages. The linoleic acid methyl ester increased from 6.04% in the green stage to 10.98% in the black stage, whereas the palmitic acid methyl ester varied between 13.4% and 17.28%. A slight decrease in palmitoleic acid methyl ester was observed, from 1.02% in the green stage to 0.33% in the black stage. Stearic acid methyl ester showed an increase directly with maturation, from 1.53% to 2.21%.

**TABLE 3:** Fatty acid composition (%) of olive oils from Akerma and Bouchouk Guergour varieties at different ripening stages (green, spotted, and black) determined by GC‐MS analysis.

Peaks	RT (min.)	Compound	Akerma (%)			Bouchouk Guergour (%)		
			Green	Spotted	Black	Green	Spotted	Black
1	47.209	Palmitoleic acid, methyl ester	1.02	0.30	0.33	0.08	0.08	0.07
2	47.723	Palmitic acid, methyl ester	17.28	13.39	14.22	9.45	9.24	9.25
3	51.708	Linoleic acid, methyl ester	6.04	10.06	10.98	5.80	6.18	6.38
4	51.857	Oleic acid, methyl ester	70.19	70.81	68.96	80.00	79.82	79.68
5	51.965	Oleic acid, methyl ester‐isomer	3.93	3.52	3.29	2.47	2.50	2.47
6	52.424	Stearic acid, methyl ester	1.53	1.92	2.21	2.22	2.19	2.15

For Bouchouk Guergour, the oleic acid methyl ester dominated all stages, ranging from 79.68% to 80.00%. Linoleic acid methyl ester increased slightly from 5.80% to 6.38%. Palmitic acid methyl ester remained stable around 9.24–9.45%, whereas the stearic acid methyl ester remained consistent at 2.15%–2.22%. These results showed that oleic acid remained the most abundant fatty acid for both varieties, with slight variations in other fatty acid content based on the maturation stage. The differences between varieties and stages highlighted the importance of maturation in determining fatty acid composition, potentially affecting the nutritional and therapeutic properties of the oils.

The GC‐MS analysis of the fatty acid composition in Akerma and Bouchouk Guergour olive oils across three maturation stages (i.e., green, spotted, and black) provides valuable insights into the dynamic changes in the fatty acid profile during ripening. Both varieties predominantly contained oleic acid methyl ester, with a relative abundance of over 68% in Akerma and 79% in Bouchouk Guergour, respectively, across all stages of maturation. This is consistent with existing literature that highlights oleic acid as the major MUFA in olive oil, which is associated with numerous health benefits, particularly cardiovascular health. Oleic acid has anti‐inflammatory properties and is known for its potential to reduce LDL cholesterol and promote heart health [26, 27].

The increase in linoleic acid methyl ester (C18:2), a polyunsaturated fatty acid (PUFA), from 6.04% in the green stage to 10.98% in the black stage in Akerma and from 5.80% to 6.38% in Bouchouk Guergour suggests that these fatty acids accumulate during ripening. Linoleic acid plays a critical role in modulating inflammatory responses, acting as a precursor to anti‐inflammatory eicosanoids [28]. This increase is important because while PUFAs like linoleic acid contribute to health benefits, they are also more prone to oxidation, which can affect the stability of the oil.

In contrast, the palmitic acid methyl ester (C16:0), a saturated fatty acid, decreased from 17.28% to 14.22% in Akerma, while it remained relatively stable in Bouchouk Guergour (fluctuating between 9.24% and 9.45%). This trend mirrors findings in other studies, where the content of saturated fatty acids, particularly palmitic acid, decreases as the fruit ripens due to the biosynthesis of more unsaturated fatty acids, such as oleic and linoleic acids. The reduction in saturated fats is considered beneficial from a health perspective, as higher levels of unsaturated fats are linked to positive health outcomes, such as lower cholesterol and reduced inflammation.

The higher content of oleic acid in Bouchouk Guergour (around 80%) compared to Akerma (approximately 70%) indicates a varietal difference in oil quality. Oleic acid is one of the key indicators of superior olive oil quality, contributing to its stability, resistance to oxidation, and health‐promoting effects [29]. The presence of oleic acid methyl ester isomers in both varieties, albeit at lower concentrations (2.47‐3.93%), further demonstrates the complexity of the fatty acid profile and its potential influence on both the oil's oxidative stability and biological activity [24].

Stearic acid methyl ester (C18:0), a saturated fatty acid, showed a slight increase with maturation in Akerma (from 1.53% to 2.21%), but remained relatively stable in Bouchouk Guergour (2.15%–2.22%). Stearic acid is known for its neutral effect on cholesterol levels, making it less detrimental compared to other saturated fatty acids [30]. The slight increase in stearic acid content in Akerma may reflect differences in fatty acid metabolism during ripening.

This study's findings are consistent with research on Mediterranean olive varieties. For example, Beltran et al. [31] reported similar trends in fatty acid composition, particularly noting the increase in unsaturated fatty acids, especially oleic acid, during olive ripening. Moreover, Tous et al. [26] corroborate the dominance of oleic acid in oils from different varieties, emphasizing its importance for both sensory characteristics and health benefits.

However, the fatty acid profile in this study, especially the higher oleic acid content in the Bouchouk Guergour variety, may also be influenced by specific environmental factors, such as soil composition, altitude, and climatic conditions, which can affect lipid metabolism in olives [28]. These factors highlight the importance of geographic and varietal differences in determining the fatty acid composition of olive oils.

### 2.5 Study of Fatty Acid by GC‐FID

**FIGURE 2:**
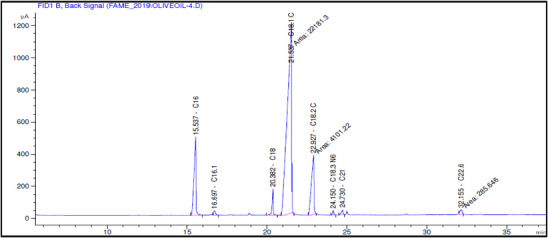
GC‐FID chromatogram showing the fatty acid composition of olive oil extracted from the Akerma variety at the green ripening stage.

**FIGURE 3:**
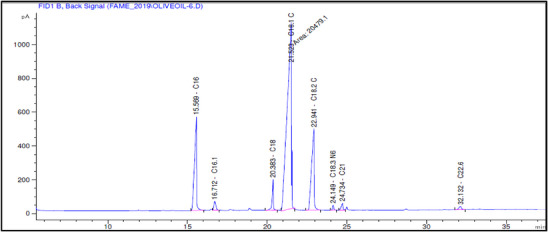
GC‐FID chromatogram showing the fatty acid composition of olive oil extracted from the Akerma variety at the spotted ripening stage.

**FIGURE 4:**
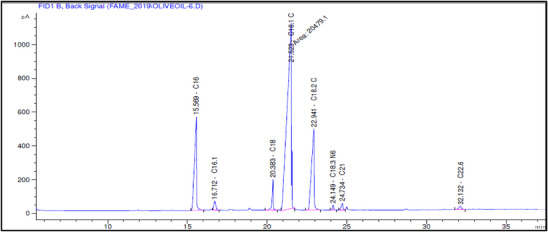
GC‐FID chromatogram showing the fatty acid composition of olive oil extracted from the Akerma variety at the black ripening stage.

**FIGURE 5:**
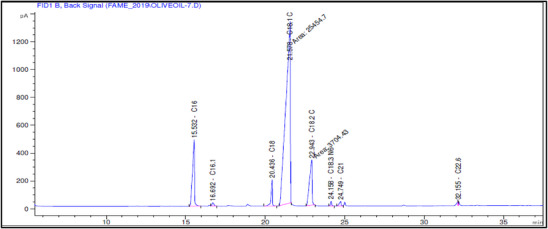
GC‐FID chromatogram showing the fatty acid composition of olive oil extracted from the Bouchouk Guergour variety at the green ripening stage.

**FIGURE 6:**
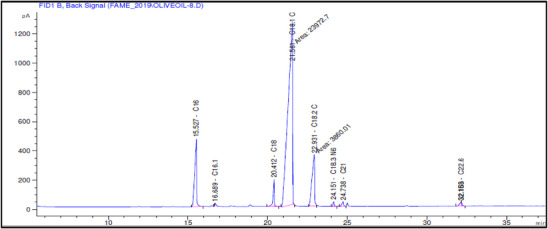
GC‐FID chromatogram showing the fatty acid composition of olive oil extracted from the Bouchouk Guergour variety at the spotted ripening stage.

**FIGURE 7:**
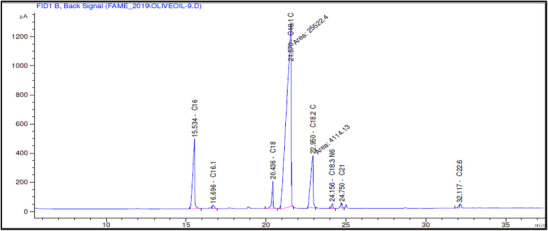
GC‐FID chromatogram showing the fatty acid composition of olive oil extracted from the Bouchouk Guergour variety at the black ripening stage.

The fatty acid composition of Akerma and Bouchouk Guergour olive oils at different maturation stages (i.e., green, spotted, and black, respectively) was analyzed with GC‐FID (Table 4, Figures 2, 3, 4, 5, 6, 7). The analysis revealed several key findings. Palmitic acid (C16:0) in Akerma increased from 12.65% at the green stage to 15.14% at the black stage, whereas in Bouchouk Guergour, the levels remained more stable, ranging from 11.24% to 11.38%. Oleic acid (C18:1 n‐9), the dominant fatty acid, was consistently higher in Bouchouk Guergour, ranging from 73.03% to 72.12%, whereas in Akerma declined from 69.30% to 62.14% with maturation. Linoleic acid (C18:2 n‐6) showed an upward trend in both varieties, particularly in Akerma, where it increased from 12.81% at the green stage to 16.41% at the black stage, whereas in Bouchouk Guergour, it ranged from 10.62% to 11.62%. Stearic acid (C18:0) remained relatively stable across maturation stages, with a range of 2.50% to 2.81% in Akerma and 2.91% to 2.96% in Bouchouk Guergour. Palmitoleic acid (C16:1) increased with maturation in Akerma, from 0.70% to 1.38%, while it remained around 0.59% in Bouchouk Guergour, with a slight dip at the spotted stage. Docosahexaenoic acid (DHA, C22:6 n‐3) was identified in small amounts, with higher levels in Akerma at the green stage (0.89%), while in Bouchouk Guergour it was significantly lower, particularly at the black stage (0.39%). These variations highlight how the maturation process influences fatty acid composition differently in the two olive varieties.

**TABLE 4:** Fatty acid composition (%) of Akerma and Bouchouk Guergour olive oils at different ripening stages determined by GC‐FID analysis.

Fatty acids found	Fatty acid groups	Akerma (%)			Bouchouk Guergour (%)		
		Green	Spotted	Black	Green	Spotted	Black
C16:0–Palmitic acid	SFA	12.65	14.46	15.14	11.38	11.24	11.35
C16:1–Palmitoleic acid	MUFA	0.70	1.22	1.38	0.59	0.20	0.58
C18:0–Stearic acid	SFA	2.50	2.59	2.81	2.96	2.91	2.95
C18:1C (n‐9)–Oleic acid ω9	MUFA/ω9FA	69.30	64.68	62.14	73.03	71.85	72.12
C18:2C (n‐6)–Linoleic acid ω6	PUFA	12.81	15.20	16.41	10.62	11.57	11.62
C18:3 (n‐6)–g‐Linolenic acid ω6	PUFA/ω6FA	0.45	0.45	0.47	0.51	0.50	0.51
C21:0–Heneicosanoic acid	SFA	0.67	0.93	0.95	0.77	0.78	0.44
C22:6 (n‐3)–cis‐4.7.10.13.16.19‐Docosahexaenoic acid (DHA)	PUFA	0.89	0.43	0.67	0.09	0.58	0.39

Abbreviations: ωFA, Omega fatty acid; MUFA, monounsaturated fatty acid; PUFA, polyunsaturated fatty acid; SFA, saturated fatty acid; TFA, Trans fatty acid.

The results revealed significant differences in the fatty acid composition between the Akerma and Bouchouk Guergour olive varieties across different maturation stages. The oleic acid (C18:1 n‐9), the dominant MUFA in olive oils, was consistently higher in Bouchouk Guergour than in Akerma. This higher oleic acid content suggests that Bouchouk Guergour has better oxidative stability and nutritional value, as oleic acid is known for its cardioprotective effects and resistance to rancidity [27, 29]. The slight decline in oleic acid during maturation, particularly in Akerma, aligns with findings by Gutiérrez et al. [30], who also observed a decrease in MUFA content as olives matured.

Linoleic acid (C18:2 n‐6), a PUFA, exhibited an opposite trend, increasing with fruit maturation, especially in Akerma. This is consistent with the work of García et al. [32], which showed that PUFAs, particularly linoleic acid, increase as olives ripen. The higher linoleic acid content in Akerma at later stages could affect the oil's stability, as PUFAs are more prone to oxidation, as noted by Tura et al. [29].

The relatively stable levels of stearic acid (C18:0) in both varieties align with typical olive oil fatty acid profiles, where stearic acid levels do not fluctuate significantly across maturation stages. According to Stefanoudaki et al. [33], the consistent presence of stearic acid supports the structural integrity of the oil's fatty acid composition and contributes to the solid fat content at cooler temperatures.

Palmitoleic acid (C16:1), although present in lower amounts, increased with maturation in the Akerma variety, potentially contributing to the flavor and sensory characteristics of the oil. The lower variability in Bouchouk Guergour suggests a more stable fatty acid profile across stages. Previous studies, such as those by Kalua and Dando [34], suggested that palmitoleic acid may enhance the sensory attributes of olive oil, especially in terms of fruity flavor.

Finally, docosahexaenoic acid (DHA, C22:6 n‐3), although present in trace amounts, was higher in the Akerma variety, particularly at the green stage. The detection of this omega‐3 fatty acid is unusual in olive oil, as noted by López‐Yerena et al. [35]. This suggests that the Akerma variety may possess unique traits that warrant further investigation. Although DHA is typically associated with marine oils, its presence, even in small amounts, could potentially add nutritional value to the oil.

These findings highlight the influence of maturation on the fatty acid composition of olive oil and provide valuable insights into optimizing the timing of harvest to maximize both the nutritional and sensory quality of the oils. The distinct fatty acid profiles of Akerma and Bouchouk Guergour varieties further suggest potential varietal differences in oxidative stability, nutritional properties, and health benefits.

### 2.6 Biological Activity Tests

#### 2.6.1 Evaluation of the Anti‐Inflammatory Activity of Olive Oils In Vivo

The anti‐inflammatory activity of olive oils extracted from *O. europaea* L. varieties Akerma and Bouchouk Guergour was evaluated at three ripening stages: green, spotted, and black, using the xylene‐induced ear edema model in mice. The inhibition percentage of edema was measured at 2 and 4 h post‐application. Comparing the results with the standard anti‐inflammatory drug. The results related to Voltaren 1% showed that the olive oils exhibited strong anti‐inflammatory effects, with inhibition rates ranging from 84.29 ± 1.24% to 92.54 ± 0.88% (Table 5).

**TABLE 5:** Percentage of inhibition of xylene‐induced ear edema in mice treated with olive oils from Akerma and Bouchouk Guergour varieties at different ripening stages (green, spotted, and black), compared with Voltaren 1% as reference drug.

Treatment	Inhibition after 2 h (%)	Inhibition after 4 h (%)
Voltaren 1%	81.63 ± 0.69^a^	79.88 ± 0.50^a^
Akerma olive oil green stage	84.29 ± 1.24^b^	85.16 ± 1.08^b^
Akerma olive oil spotted stage	87.69 ± 1.20^c^	89.32 ± 1.14^c^
Akerma olive oil black stage	90.31 ± 0.80^d^	91.09 ± 0.80^d^
Bouchouk Guergour olive oil green stage	89.52 ± 0.95^c^ ^d^	89.78 ± 1.10^c^
Bouchouk Guergour olive oil spotted stage	86.91 ± 0.93^c^	87.36 ± 0.90^b^ ^c^
Bouchouk Guergour olive oil black stage	92.14 ± 0.72^e^	92.54 ± 0.88^e^

*Note*: Values are expressed as mean ± SD (*n* = 6). Different letters indicate statistically significant differences between treatments (*p* < 0.05).

For the Akerma variety, the oil from the black ripening stage showed the highest inhibition rate, 91.09 ± 0.80% after 4 h. The spotted‐stage oil also demonstrated high efficacy, with 89.32 ± 1.14% inhibition, whereas the green‐stage oil showed slightly lower activity, of 85.16 ± 1.08%. Similarly, for the Bouchouk Guergour variety, the black‐stage oil exhibited the highest anti‐inflammatory effect, with 92.54 ± 0.88% inhibition after 4 h, followed by the spotted and green stages, each with 87.36 ± 0.90% and 89.78 ± 1.10%, respectively. Notably, both black‐stage oils of Akerma and Bouchouk Guergour varieties outperformed the reference drug Voltaren, which achieved an inhibition rate of 79.88 ± 0.50% after 4 h. For the Akerma variety, the oil from the black stage demonstrated the highest anti‐inflammatory activity, with 91.09 ± 0.80% inhibition after 4 h. The oils from the spotted and green stages showed slightly lower but still significant inhibition rates (89.32 ± 1.14% and 85.16 ± 1.08%, respectively). The Bouchouk Guergour variety oil from the black stage also demonstrated the highest efficacy, with 92.54 ± 0.88% inhibition after 4 h. The oils from the green and spotted stages followed closely, with inhibition rates of 89.78 ± 1.10% and 87.36 ± 0.90%, respectively.

Statistical analysis revealed significant differences in the anti‐inflammatory activity between treatments at both 2 and 4 h post‐application (*p* < 0.05). Olive oils obtained at the black ripening stage from both Akerma and Bouchouk Guergour varieties exhibited significantly higher edema inhibition compared to Voltaren 1% and to oils extracted at earlier ripening stages.

The anti‐inflammatory activity of Akerma and Bouchouk Guergour olive oils can be closely linked to their fatty acid composition, as revealed by the GC‐MS and GC‐FID analyses. Oleic acid (C18:1 n‐9) is the most prominent fatty acid in both olive varieties, with concentrations of 69.30% in Akerma and 73.03% in Bouchouk Guergour at the green stage. Numerous studies have reported the important role played by oleic acid in reducing inflammation through various mechanisms, including the modulation of inflammatory cytokines. For instance, oleic acid has been shown to downregulate the expression of pro‐inflammatory markers such as tumor necrosis factor‐alpha (TNF‐α) and interleukin‐6 (IL‐6), thereby mitigating inflammatory responses in various models [35].

In addition to oleic acid, linoleic acid (C18:2 n‐6), which increased from 12.81% in Akerma variety at the green stage to 16.41% at the black stage, also contributes to the anti‐inflammatory properties of these oils. Linoleic acid is a precursor in the synthesis of anti‐inflammatory eicosanoids, such as prostaglandin E1 (PGE1), which can counteract the effects of pro‐inflammatory eicosanoids. The elevation of linoleic acid during maturation suggests that Akerma and Bouchouk Guergour oils may possess enhanced anti‐inflammatory potential at later maturation stages, aligning with findings from Tura et al. [29], which underlined the importance of linoleic acid in reducing chronic inflammation.

Furthermore, the palmitoleic acid (C16:1), although present in smaller amounts, was also associated with anti‐inflammatory activities. Recent research indicates that palmitoleic acid can reduce the expression of inflammatory cytokines and modulate lipid profiles, which may contribute to its protective effects against inflammatory diseases [30]. The increase in palmitoleic acid in Akerma during maturation indicates a potential enhancement in the oil's anti‐inflammatory capabilities, adding another layer to its health benefits.

Overall, the combination of these fatty acids suggests that Akerma and Bouchouk Guergour olive oils possess significant anti‐inflammatory properties, making them valuable additions to a health‐conscious diet. The unique fatty acid profiles highlight their potential in managing inflammation‐related conditions, offering a natural and dietary approach to promoting overall health.

##### 2.6.1.1 Mechanistic Explanation for Black‐Stage Olive Oil's Better Anti‐Inflammatory Effects

The enhanced anti‐inflammatory effects of the black‐stage olive oils are likely due to the increased concentrations of oleic acid and polyphenols, which are known for their anti‐inflammatory properties. Oleic acid, the predominant MUFA in olive oil, has been shown to reduce LDL cholesterol and modulate pro‐inflammatory cytokines, promoting heart health and reducing inflammation [27]. Additionally, polyphenols such as oleuropein and hydroxytyrosol, which accumulate at later stages of ripening, possess strong antioxidant properties that protect against oxidative stress, a key driver of inflammation [19]. This biochemical profile is likely the reason for the superior anti‐inflammatory activity observed in the black‐stage oils.

#### 2.6.2 Evaluation of the Wound Healing Activity of *O. europaea* L. Olive Oil In Vivo

Table 6 presents the evaluation of wound surface area (in mm^2^) in rats over a 28‐day treatment period, offering critical insights into the efficacy of various treatments on wound healing processes. Initial observations indicate that on the first day, the wounds across all treatment groups exhibited relatively similar sizes, with Bouchouk Guergour oil in the green stage showing the largest wound area (i.e., 398.32 mm^2^). This suggests a consistent baseline before the initiation of treatments. As treatment progressed, significant differences in wound healing effectiveness emerged. For instance, by Day 28, Bouchouk Guergour and Akerma olive oils, particularly in their black stages, demonstrated substantial reductions in wound size, ultimately achieving complete healing (0 mm^2^). The control treatment, Vaseline, revealed less effectiveness in reducing wound size than the olive oil treatments. The presence of mortality in the negative control raises safety concerns, emphasizing the need for appropriate treatment selection. Additionally, the observed contraction percentages further reflected healing effectiveness, supporting the efficacy of olive oils. Overall, the findings indicate that Bouchouk Guergour and Akerma olive oils, especially in their black stages, were highly effective therapeutic agents for wound management, as evidenced by the significant reduction in wound surface area over time.

**TABLE 6:** Evolution of wound surface area (mm^2^) in rats treated with olive oils from Akerma and Bouchouk Guergour varieties at different ripening stages over a 28‐day experimental period, compared with positive and negative control treatments.

Day	Vaseline	Negative control	Positive control (Biafine)	Bouchouk Guergour			Akerma		
				Green	Spotted	Black	Green	Spotted	Black
D1	289.91^a^	282.82^a^	292.87^a^	398.32^a^	319.65^a^	358.91^a^	383.21^a^	344.44^a^	346.16^a^
D4	323.11^a^	290.26^a^	273.34^a^	542.17^b^	348.85^a^	404.08^b^	429.94^b^	468.10^b^	359.28^a^
D7	324.37^a^	291.43^a^	291.29^a^	454.80^b^	334.15^a^	546.58^c^	541.53^c^	374.15^a^	326.14^a^
D10	311.05^a^	292.20^a^	261.85^a^ ^b^	311.34^a^	201.01^b^	328.11^a^	453.99^c^	310.42^a^	302.76^a^
D13	324.80^a^	354.16^a^	253.54^b^	205.11^b^	119.37^c^	164.13^c^	350.79^a^	210.80^b^	234.55^b^
D16	311.42^a^	350.31^a^	191.61^b^	132.87^c^	100.38^c^	56.86^d^	256.02^b^	126.51^c^	130.95^c^
D19	271.98^a^	Death	116.59^b^	82.49^c^	63.36^c^	27.20^d^	157.95^b^	80.61^c^	72.67^c^
D22	92.13^a^	Death	61.24^b^	57.72^b^	32.61^c^	4.505^d^	110.61^a^	44.40^c^	13.92^d^
D25	84.73^a^	Death	38.75^b^	5.65^c^	25.01^b^	0^d^	81.72^a^	13.42^c^	7.30^c^
D28	83.7^a^	Death	26.24^b^	0^c^	0^c^	0^c^	72.27^a^	0^c^	3.98^c^

*Note*: The different letters indicate statistically significant differences.

Statistical analysis revealed significant differences in wound surface area between treatments at each experimental time point from Day 7 onward (*p* < 0.05). Rats treated with olive oils obtained at the black ripening stage of both Akerma and Bouchouk Guergour varieties exhibited significantly faster wound contraction compared to positive and negative control groups, leading to complete wound closure by Day 28.

Table 7 analyzes the percentage of wound healing for various treatments over the same 28‐day period, offering further insight into treatment effectiveness. Initial healing percentages reveal that all treatments except positive control (Biafine) displayed negative values at Day 4, indicating that wounds either worsened or did not improve, possibly due to inflammatory responses. However, by Day 10, some treatments began to show positive healing, particularly the black stage of Bouchouk Guergour and Akerma oils. Notably, by Day 16, the healing percentages significantly increased, reaching 66.64% for Bouchouk Guergour and 68.60% for Akerma, reflecting substantial improvements in wound healing capabilities. Remarkably, both treatments achieved 100% healing by Day 28, suggesting the presence of potent bioactive compounds that facilitate and accelerate the healing process. In contrast, the negative control demonstrated complete failure in healing, underscoring the importance of using effective therapeutic agents. Furthermore, the observed contraction percentages relative to the initial wound sizes highlighted the effectiveness of olive oil treatments, whereas higher percentages correlated with improved healing outcomes.

**TABLE 7:** Percentage of wound contraction in rats treated with olive oils from Akerma and Bouchouk Guergour varieties at different ripening stages during the 28‐day healing period.

Day	Vaseline%	Negative control %	Positive control (Biafine) %	Bouchouk Guergour %			Akerma %		
				Green	Spotted	Black	Green	Spotted	Black
D4	−11.43^a^	−2.63^a^	6.66^b^	−36.14^c^	−9.13^a^	−12.59^a^	−12.16^a^	−35.87^c^	−3.79^a^
D7	−11.87^a^	−3.05^a^	0.54^b^	−14.19^a^	−4.54^a^	−52.29^c^	−41.34^c^	−8.63^a^	5.79^a^
D10	−7.26^a^	−3.32^a^	10.59^b^	21.82^c^	37.12^c^	8.58^b^	−18.49^a^	9.88^a^	12.53^b^
D13	−12.02^a^	−25.27^c^	13.43^b^	48.48^d^	62.65^e^	54.27^c^	8.46^b^	38.80^c^	32.24^c^
D16	−7.41^a^	−23.88^c^	34.57^b^	66.64^c^	68.60^c^	84.16^d^	33.19^b^	63.26^c^	62.17^c^
D19	6.19^a^	Death	60.20^b^	79.29^c^	80.18^c^	92.42^d^	58.77^b^	76.59^c^	79.01^c^
D22	68.21^a^	Death	79.09^b^	85.58^c^	89.79^c^	98.87^d^	71.14^a^	87.10^c^	96.12^d^
D25	70.76^a^	Death	86.77^b^	98.58^c^	92.17^c^	100.00^c^	78.68^a^	96.10^c^	97.89^c^
D28	71.14^a^	Death	91.04^b^	100.00^c^	100.00^c^	100.00^c^	81.13^a^	100.00^c^	98.85^c^

*Note*: These percentages show the contraction of wounds relative to the initial size (Day 0) on Day 1. When the rate is high, it indicates good healing, but when it is negative, it indicates worsening or an increase in the wound. The different letters indicate statisticalle significant differences.

From Day 10 onward, statistically significant differences in wound healing were observed between treatment groups (p < 0.05). Rats treated with olive oils from the black ripening stage of both Akerma and Bouchouk Guergour varieties showed significantly faster wound contraction and complete healing compared to control treatments.

The data from Table 7 strongly support that the olive oils extracted from both varieties, particularly in their black stages, were highly effective in promoting wound healing, thus reinforcing their potential as natural therapeutic alternatives for wound management.

Different superscript letters within the same row indicate statistically significant differences between treatments at the same time point (one‐way ANOVA followed by Tukey's post hoc test, *p* < 0.05).

Negative values indicate wound enlargement, whereas positive values indicate wound contraction. Mortality was observed in the negative control group from Day 19 onward.

Statistical analysis of wound contraction percentages showed significant differences between treatments at each experimental time point from Day 10 onward (*p* < 0.05). Olive oils obtained at the black ripening stage from both Akerma and Bouchouk Guergour varieties exhibited significantly higher wound contraction rates compared to control treatments, achieving complete healing by Day 28.

The values from Tables 6 and 7 highlight the remarkable wound‐healing efficacy of Bouchouk Guergour and Akerma olive oils, particularly in their black stages. The initial observations indicated a consistent baseline for wound sizes across treatment groups on Day 1. However, as the treatment progressed, significant differences emerged in the healing capabilities of the various treatments, with both olive cultivars demonstrating superior performance. By Day 28, the complete healing of wounds treated with Bouchouk Guergour and Akerma oils underscored their potent therapeutic properties.

Comparative studies showed that the bioactive compounds in olive oils, such as oleuropein and hydroxytyrosol, play critical roles in enhancing wound healing through anti‐inflammatory and antioxidant mechanisms [36, 37]. These findings are consistent with other research that emphasizes the importance of plant‐based treatments in promoting wound recovery. For example, the study of Owen et al. [38] reported that topical applications of olive oil significantly improved healing outcomes compared to conventional treatments, further supporting the observed benefits in this study.

Moreover, the poor healing outcomes and associated mortality of the negative control group highlight the necessity of selecting effective therapeutic agents for wound management. In contrast, the significant healing percentages recorded for the olive oil treatments indicate they are safe and potent alternatives to synthetic treatments. The healing percentages reflect a direct correlation between the contraction of wound surface area and the treatment effectiveness, aligning with research indicating that higher contraction rates were indicative of enhanced healing outcomes [39].

## 3 Conclusions

The comprehensive analysis of the ripening dynamics, oil yield, and physicochemical characteristics of the Akerma and Bouchouk Guergour olive varieties revealed crucial insights into their maturation processes and quality. The ripening index indicated that, whereas both varieties progressively matured, the Bouchouk Guergour displayed a faster maturation trajectory, allowing it to reach an advanced ripening stage at a lower index. This rapid maturation may confer advantages in regions with shorter growing seasons, enabling early harvesting to capture desirable oil attributes. Oil yield patterns further illustrated the distinct behaviors of these varieties. The Bouchouk Guergour variety achieved a peak yield of 19.56 ± 0.22% at full ripeness, whereas Akerma reached its maximum yield of 12.71 ± 0.17% at the spotted stage. These results align with established patterns observed in other global olive varieties, emphasizing the role of genetic and environmental factors in oil accumulation dynamics. The physicochemical analysis highlighted the importance of free acidity, peroxide value, water content, iodine value, and fatty acid composition in determining olive oil quality. Both varieties adhered to International Olive Council standards, with Akerma exhibiting lower acidity and Bouchouk Guergour showing higher oleic acid content. The changes in fatty acid profiles during maturation, particularly the increase in linoleic acid and the predominance of oleic acid methyl ester reflect the unique biochemical pathways each variety undergoes. In addition to these findings, the evaluation of the wound‐healing activity of olive oil in vivo demonstrates the therapeutic potential of these oils. The significant reduction in wound surface area and the high percentages of healing observed in treatments using Bouchouk Guergour and Akerma olive oils suggest that these oils possess bioactive compounds that facilitate the healing process. The results underscore the need for tailored harvest strategies based on varietal characteristics and ripening stages to optimize oil yield and quality. This research can guide olive oil producers in optimizing their harvest strategies, such as determining the ideal ripening stage for maximum oil yield and quality, and adjusting harvesting practices based on regional growing conditions. Future research focusing on the long‐term stability and bioavailability of olive oil components, as well as the interplay of climatic conditions and varietal traits, will provide deeper insights into maximizing the potential of these olive varieties in the context of sustainable agricultural practices. These findings hold practical significance for multiple stakeholders. Local olive producers can utilize the data to tailor harvesting strategies according to the specific characteristics of each cultivar, maximizing both yield and quality. The Algerian olive oil industry may leverage these insights to promote high‐value functional oils with enhanced therapeutic properties. Additionally, the research community can use these results as a foundation for applied studies focusing on the bioavailability and long‐term stability of active compounds in olive oils.

## 4 Experimental Section

### 4.1 Plant Material

**FIGURE 8:**
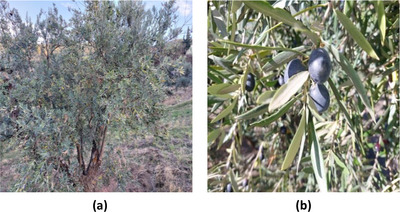
(a) Olive tree of the Bouchouk Guergour variety and (b) corresponding olive fruits collected at different ripening stages. All images are original and were created by the authors for this manuscript.

**FIGURE 9:**
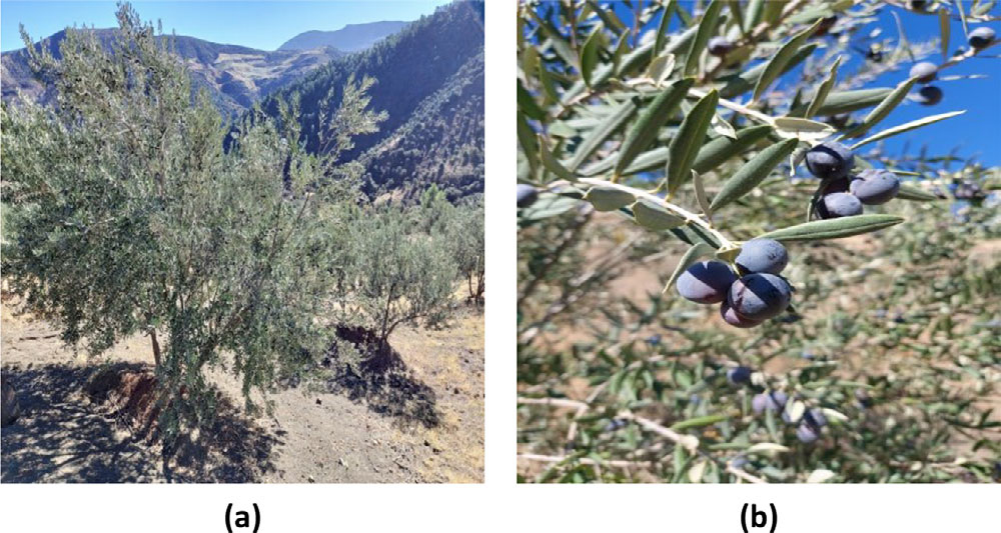
(a) Olive tree of the Akerma variety and (b) corresponding olive fruits collected at different ripening stages. All images are original and were created by the authors for this manuscript.

Two olive varieties, namely, Akerma and Bouchouk Guergour (Figures 8 and 9), were cultivated in Draâ Kebila, a municipality in the Sétif Province of Northeastern Algeria. The area is situated in the Tell Atlas Mountain, at an elevation of 1100 to 1200 m, and is characterized by a semi‐arid Mediterranean climate with hot summers and cool, wet winters. These varieties were selected based on prior knowledge of local olive orchards, known for their high‐quality oil production.

The olive trees were cultivated under traditional agronomic practices in non‐irrigated fields with calcareous‐clay soils. Organic fertilization was applied once a year using decomposed livestock manure during the autumn season. The trees were approximately 25 years old, planted with a spacing of 6 × 6 m. For each variety, fruit samples were collected from 10 randomly selected healthy trees, each yielding an average of 15 to 20 kg of fruit. These standardized conditions aimed to minimize environmental variability and ensure reliable comparisons.

Olive samples were collected in the rural commune of Draâ Kebila, with trees randomly selected from similar pedoclimatic conditions. For each variety, three sampling sessions were conducted between October and December 2022. The plant material was carefully chosen, as such as to ensure that the tree was healthy, vigorous, free of diseases and pests, as well as untreated.

The olive trees were cultivated in the Sétif region, characterized by a semi‐arid Mediterranean climate. The area receives moderate annual rainfall, averaging approximately 480 mm, with relative humidity ranging from 38% to 52%. The region lies at an elevation of 1100 to 1200 m above sea level. The soil is predominantly calcareous‐clay, which, combined with limited water availability, influences the biochemical profile of the olives [40].

### 4.2 Plant Material

Olive fruits (*O. europaea* L.) belonging to the Akerma and Bouchouk Guergour cultivars were collected during the 2022 harvest season from orchards located in Draâ Kebila (Sétif region, northeastern Algeria; 36°13′ N, 5°40′ E). Fruits were harvested manually at three ripening stages (green, spotted, and black) based on skin color and ripening index.

The plant material was botanically identified by Pr. Amar Benmahammed, Department of Plant Biology and Physiology, Ferhat Abbas University Sétif 1, Algeria. Voucher specimens were prepared and deposited in the Herbarium of the Faculty of Natural and Life Sciences, Ferhat Abbas University Sétif 1, under the following accession numbers: Akerma (Voucher No. OA‐AK‐2022) and Bouchouk Guergour (Voucher No. OA‐BG‐2022).

### 4.3 Optimal Harvest Stage

The optimal harvest period was determined following Uceda and Hermoso [11]. One hundred olives were randomly selected from a 1 kg batch, based on the color of the fruit's skin and flesh, as defined by the International Olive Council [41].

The index varies from 0 to 7 and is calculated using the following formula:

$$\begin{array}{ccc} \mathrm{IM} & = & [\left(0n0\right)+\left(1n1\right)+\left(2n2\right)+\left(3n3\right)+\left(4n4\right)+\left(5n5\right)+\left(6n6\right)\\ & & +\left(7n7\right)]/100 \end{array}$$

Where:

- n0, n1, n2, n3, n4, n5, n6, and n7 represent the number of olive fruits in the following eight categories.
- Index 0: Olive with intense green or dark green skin.
- Index 1: Olive with yellow or yellowish‐green skin.
- Index 2: Olive with yellowish skin showing reddish spots or areas.
- Index 3: Olive with reddish or light purple skin.
- Index 4: Olive with black skin and entirely green pulp.
- Index 5: Olive with black skin and purple pulp up to half its thickness.
- Index 6: Olive with black skin and purple pulp down to the pit.
- Index 7: Olive with black skin and completely dark pulp.

### 4.4 Olive Oil Extraction Method

**FIGURE 10:**
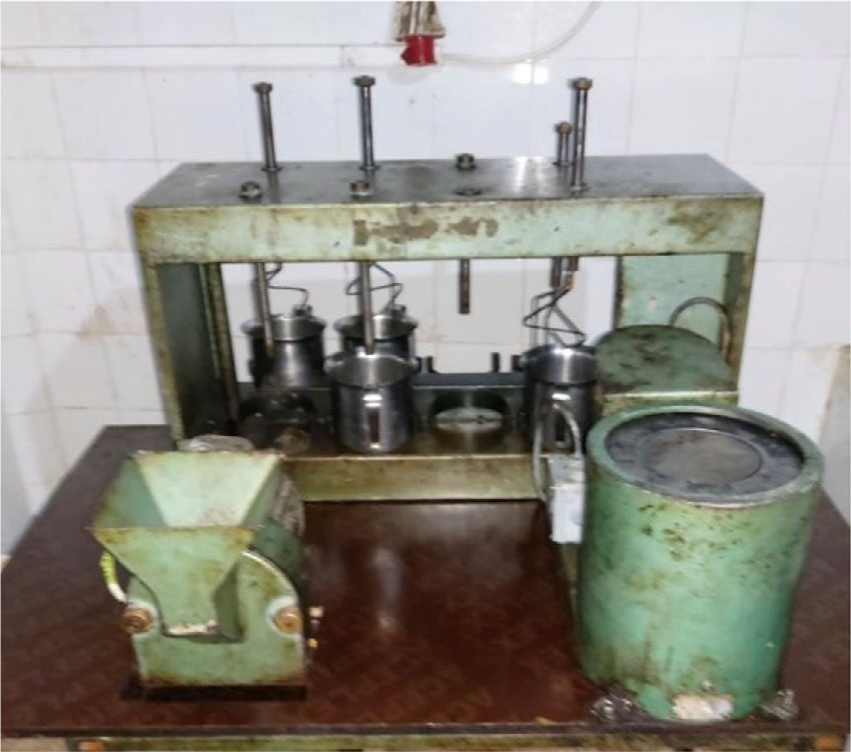
Oil extraction system (oleodoseur) used at the Technical Institute of Fruit Arboriculture and Vineyards (TIFAV), Sidi‐Aiche, Algeria. All images are original and were created by the authors for this manuscript.

Oil extraction was carried out at the Technical Institute of Fruit Arboriculture and Vineyards (TIFAV) laboratory in Sidi‐Aiche using an oil doser (Figure 10).

**FIGURE 11:**
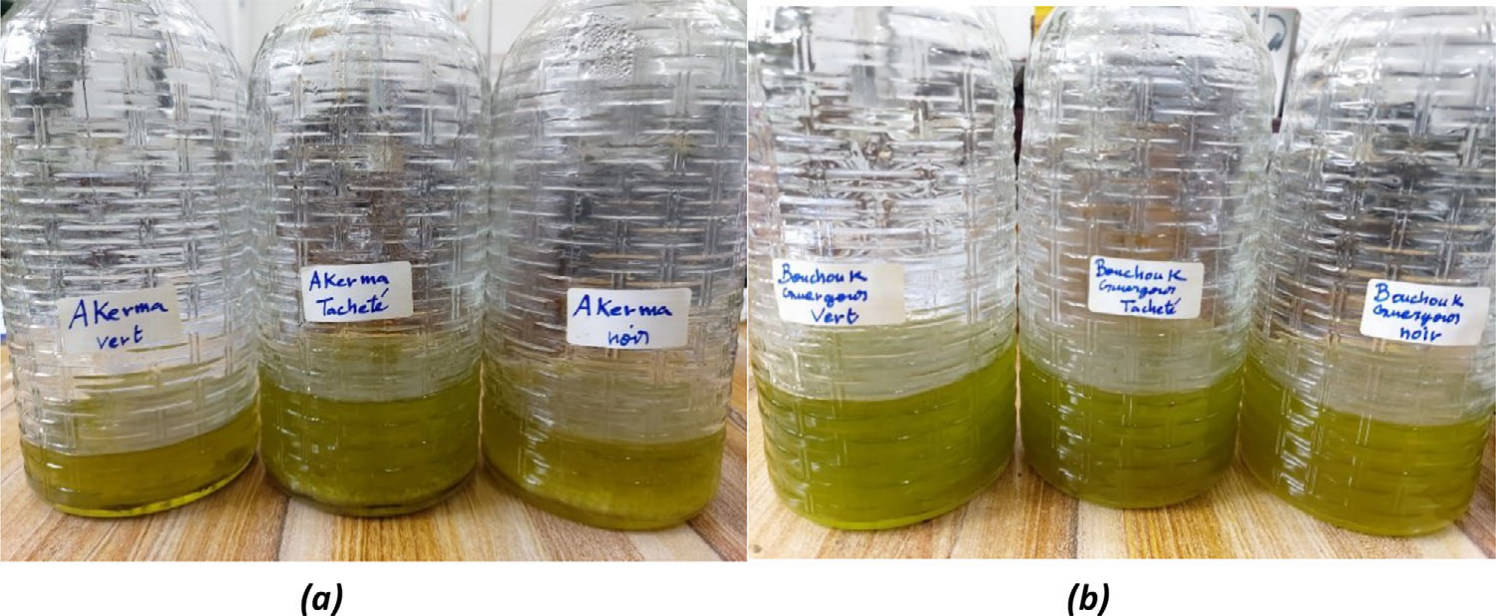
Olive oil obtained after decantation from (a) Akerma and (b) Bouchouk Guergour varieties at green, spotted, and black ripening stages. All images are original and were created by the authors for this manuscript.

The extraction process involved the following steps:

- Crushing: the olives were crushed with a mechanical hammer mill to fine, homogeneous paste.
- Malaxation: The olive paste was kneaded (malaxation) in a stainless‐steel vat for 20 min at a controlled temperature of 25°C, following the protocol established by Clodoveo et al. [42], which has been shown to optimize the extraction of bioactive compounds while preserving oil quality.
- Centrifugation: The paste was transferred to a centrifuge with an open cylindrical bowl and a liquid outlet, operating at 3000 RPM. The oil and vegetable water were collected in a container.
- Decantation: The separation of oil from vegetable water was achieved by exploiting density differences. The oil, with a lower density of 0.92, rose to the surface and was easily collected (Figure 11).

After extraction, the olive oil was immediately filtered through Whatman No. 1 filter paper to remove solid residues and residual moisture. The filtered oils were then stored in amber glass bottles at 4 °C, protected from light, until further analysis. All analytical procedures were performed within 72 h following oil extraction to minimize oxidative degradation and preserve the native biochemical composition. The extracted olive oil samples were stored in amber glass bottles, tightly sealed, and kept at 4 °C in the dark to prevent oxidation and degradation of sensitive compounds prior to analysis.

### 4.5 Animal Material

**FIGURE 12:**
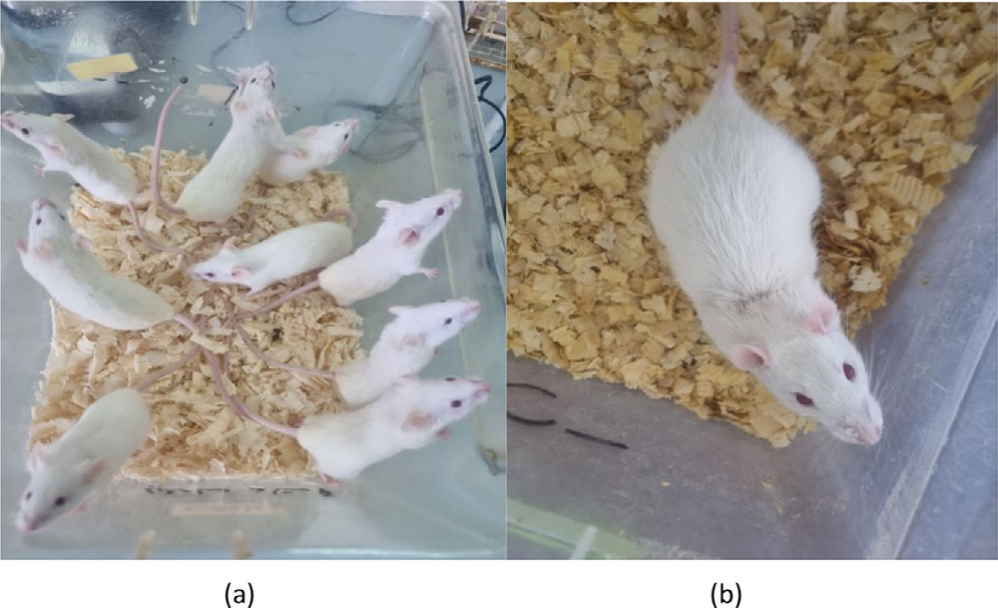
Experimental animal models used for biological activity assays: (a) Swiss albino mouse and (b) Wistar rat. All images are original and were created by the authors for this manuscript.

In the current study, two animal species were used: Swiss albino mice, of 22–36 g mass, and Wistar rats, of 250–300 g mass (Figure 12). Both species of rodents were provided by the Pasteur Institute of Algiers and were used for in vivo tests. The experimental protocol was approved by the ethics committee of the Faculty of Natural and Life Sciences at Ferhat Abbas University, Sétif 1. This approval ensures that the current study adhered to the ethical standards concerning the treatment and care of animals, as outlined in the Declaration of Helsinki and the guidelines set forth by the International Council for Laboratory Animal Science (ICLAS). These ethical frameworks emphasize the importance of minimizing the pain, distress, and suffering of the animals involved in research. The committee reviewed the study design as such as to confirm that all procedures were conducted in compliance with the principles of the 3Rs (Replacement, Reduction, and Refinement), aiming to reduce the number of animals used and enhance their welfare. The animals were housed in plastic cages with free access to food and water throughout the experiments. The cages were cleaned every 3 days. The maintenance conditions in the animal facility were as follows:

- Ambient temperature: 19 to 22°C
- Humidity: 38% to 52%
- Lighting: 12 h of light per day
- Water: Tap water
- Food: Pellets (Provided by the National Animal Feed Board ‐ NAFB)
- Light/dark cycle: 12H/12H

All animal experiments were conducted in accordance with international guidelines for the care and use of laboratory animals. The experimental protocols were approved by the Institutional Ethics Committee of the Faculty of Natural and Life Sciences, Ferhat Abbas University Sétif 1, Algeria (Approval No. CE‐FSNV‐2022‐017).

Male Wistar rats (180–220 g) and Swiss albino mice were obtained from the animal facility of the Faculty of Natural and Life Sciences. All efforts were made to minimize animal suffering and to reduce the number of animals used.

### 4.6 Oil Yield Calculation

The oil yield was determined by comparing the volume of oil extracted to the initial mass of the used olives. It is expressed as a percentage and calculated using the following formula:

$$\begin{array}{ccc} \mathrm{Oil}\;\mathrm{Yield}\;\left(\%\right) & = & \left[\mathrm{Volume}\;\mathrm{of}\;\mathrm{oil}\;\mathrm{extracted}\;\left(\mathrm{mL}\right)/\right.\\ & & \left.\mathrm{Initial}\;\mathrm{weight}\;\mathrm{of}\;\mathrm{olives}\;\left(g\right)\right]\times 100. \end{array}$$

### 4.7 Physicochemical Analysis

#### 4.7.1 Free Acidity Measurements

Free acidity is expressed as a percentage of oleic acid and is measured by titrating 1 g of olive oil in 50 mL of a solvent mixture primarily composed of diethyl ether, in accordance with the International Olive Council protocol (IOC, 2017) [43]. In certain steps, distilled water was added during phase separation to ensure the removal of residual acidic or basic impurities. The titration is performed using a 0.1N potassium hydroxide (KOH) solution. During titration, 0.3 mL of 1% phenolphthalein solution in ethanol is used as an indicator, and the endpoint is indicated by the appearance of a pale pink coloration. The free acidity is then calculated using the following formula:

$$\begin{array}{ccc} \mathrm{Free}\;\mathrm{acidity}\;\left(\%\right) & = & \left[\mathrm{Volume}\;\mathrm{of}\;\mathrm{KOH}\;\left(\mathrm{mL}\right)-\mathrm{Normality}\;\mathrm{of}\;\mathrm{KOH}\right.\\ & & \left.\times \;28.2\right]/\mathrm{weight}\;\mathrm{of}\;\mathrm{oil}\;\mathrm{sample}\;\left(g\right) \end{array}$$

#### 4.7.2 Peroxide Value Measurements

The peroxide value was determined using the acetic acid and chloroform method as described by ISO 3960 (2007). In this procedure, 1 g of olive oil was dissolved in 12.2 mL of acetic acid and chloroform mixture (3:2 ratio, v/v). To this, 15 mL of saturated potassium iodide (KI) solution was added, and the mixture was kept in the dark for 5 to 10 min. Afterward, 60 mL of distilled water and 1 mL of starch solution were added, resulting in the appearance of purple color. The mixture was then titrated with 0.01N sodium thiosulfate solution (Na_2_S_2_O_3_) until the purple color disappeared. The peroxide value, expressed in milliequivalents of oxygen per kilogram of oil (meq O_2_/kg), was calculated based on the following formula (ISO 3960, 2007):

$$\mathrm{PV}=\left(V-\mathrm{Vo}\right)\times N\times 1000/m$$

where:

- *V* = volume of Na_2_S_2_O_3_ solution used for the titration (mL),
- *V*_0_ = volume of Na_2_S_2_O_3_ solution used for the blank (mL),
- *N* = normality of the Na_2_S_2_O_3_ solution (0.01 N),
- *m* = mass of the oil sample (g).

#### 4.7.3 Water and Volatile Matter Content Measurements

The measurement of water and volatile matter content was conducted according to the AFNOR NF T60‐201 (1984) method, which in turn is based on the principle of evaporating water by placing a known quantity of oil in an oven maintained at 103°C for 30 min. The results were expressed as percentages by mass and calculated using the following formula (AFNOR NF T60‐201, 1984):

$$\begin{array}{ccc} H\left(\%\right) & = & \left[\mathrm{Initial}\;\mathrm{mass}\;\mathrm{of}\;\mathrm{sample}-\mathrm{Mass}\;\mathrm{after}\;\mathrm{heating}\right]/\\ & & \mathrm{Initial}\;\mathrm{mass}\;\mathrm{of}\;\mathrm{sample} \end{array}$$

#### 4.7.4 Iodine Value Measurement

The iodine value was determined by measuring the degree of unsaturation of the fatty acids according to NF T60‐203 (1990). A known amount of oil was dissolved in chloroform and reacted with the excess of iodine monochloride (Wij's solution). The consumed iodine by the unsaturated fatty acids was subsequently titrated with sodium thiosulfate to calculate the iodine value. The iodine value was expressed as the number of g of iodine absorbed per 100 g of oil, indicating the unsaturation (NF T60‐203, 1990).

#### 4.7.5 Biochemical Analysis

Both GC‐MS and GC‐FID were used in this study to provide a more comprehensive analysis of the olive oil's fatty acid and volatile compound composition. GC‐MS offers high sensitivity and specificity for identifying complex, low‐concentration volatile compounds, and its mass spectra enable precise compound identification and structural characterization. On the other hand, GC‐FID is more commonly used for quantifying fatty acid compositions, offering excellent reproducibility and high sensitivity for detecting fatty acids and other organic compounds. By combining these two methods, we gain both qualitative (GC‐MS) and quantitative (GC‐FID) data, which allows for a more thorough and reliable analysis of the olive oil composition.

#### 4.7.6 Analysis of Fatty Acid by SPME and GC‐MS

The analysis of volatile compounds in olive oil was performed with SPME as well as with GC‐MS [44]. Two‐gram samples of olive oil were placed in 10 mL vials and sealed with silicone septums. Internal standards, specifically 4‐methyl‐2‐pentanone (1.5 g), were added to the samples. The mixtures were then pre‐incubated at 40°C for 10 min. Volatile compounds from the headspace were adsorbed onto a 75 µm divinylbenzene/carboxen‐polydimethylsiloxane (PDMS) SPME fiber for 30 min. This was followed by separation using gas chromatography and analysis via mass spectrometry. The analysis was conducted using an Agilent 6890 N Network apparatus, with a ZB‐WAX capillary column (3 mm internal diameter, 1 µm film thickness). The column was initially maintained at 40°C for 10 min and then heated at 3°C per minute until it reached 200°C. Mass spectra were recorded with electron impact ionization at 70 eV, covering a mass range from 15 to 250 atomic mass units.

This dual technique of GC‐MS provides a comprehensive analysis of volatile compounds, as GC‐MS offers both qualitative and quantitative data, enabling the identification of volatile compounds at low concentrations and the structural elucidation of complex mixtures. GC‐MS is particularly valuable for the analysis of volatile compounds due to its sensitivity and ability to provide mass spectra for precise identification.

#### 4.7.7 Analysis of Fatty Acid by GC‐FID

The fatty acid composition of olive oil was determined using gas chromatography‐flame ionization detection (GC‐FID). Methyl esters of the olive oil were prepared following the method by Kyriakidis and Dionysopoulos [45] and the International Olive Council [43]. A 0.2 g sample of olive oil was esterified by adding 2 mL of hexane and 0.2 mL of 1N KOH in methanol. The mixture was shaken thoroughly and left in the dark to allow phase separation. The clear upper phase was then transferred to vials for analysis. GC‐FID was performed using an Agilent 7820 A system, equipped with SP 2560 columns (100 m × 0.25 mm × 0.2 µm). A 1 µL injection volume was used with a split injection ratio of 1:20, and hydrogen was the carrier gas at a linear velocity of 41 cm/s. The split flow rate was set at 400 mL/min, and the detector temperature was maintained at 260°C. The oven temperature program began at 100°C (held for 5 min), then increased to 240°C at a rate of 4°C/min (held for 20 min). A secondary program started at 120°C (held for 1 min), then increased to 175°C at a rate of 10°C/min (held for 10 min), followed by an increase to 210°C at a rate of 5°C/min (held for 5 min), and finally to 230°C at 5°C/min (held for 5 min), with a total runtime of 37.75 min.

The Supelco 37 Component FAME Mix (Sigma‐Aldrich) was used as a reference standard for quantification. The GC‐FID method is widely used for the analysis of fatty acids because it provides high sensitivity, reproducibility, and is capable of distinguishing between fatty acids based on their carbon chain lengths and double bond positions. The use of FID ensures that any organic compound with a carbon‐hydrogen bond can be detected with high sensitivity, making it ideal for analyzing the fatty acid profiles of oils.

### 4.8 Biological Activity Tests

#### 4.8.1 Assessment of the Anti‐Inflammatory Activity of Olive Oils In Vivo

The protocol for measuring the anti‐inflammatory effects of olive oils was conducted in vivo. To investigate the anti‐edematous effects of oils extracted from the Akerma and Bouchouk Guergour varieties, at three different ripening stages (i.e., green, spotted, and black, respectively), the method of Mayouf et al. [46] was used. This in vivo test aimed to assess the anti‐inflammatory properties of the six olive cultivars using the ear edema model, which was induced by the topical application of xylene.

The experiment involved 40 mice, which were divided into 8 groups of 5 individuals each, as outlined below.

- Group 1: Negative control group, comprising untreated mice.
- Group 2: Positive control group, where the mice was treated with 20 µL of Voltaren 1% cream on the inner surface of the right ear.
- Group 3: mice treated with 20 µL of olive oil extracted from the Akerma variety, green stage.
- Group 4: mice treated with 20 µL of olive oil extracted from the Akerma variety, spotted stage.
- Group 5: mice treated with 20 µL of olive oil extracted from the Akerma variety, black stage.
- Group 6: mice treated with 20 µL of olive oil extracted from the Bouchouk Guergour variety, green stage.
- Group 7: mice treated with 20 µL of olive oil extracted from the Bouchouk Guergour variety, spotted stage.
- Group 8: mice treated with 20 µL of olive oil extracted from the Bouchouk Guergour variety, black stage.

**FIGURE 13:**
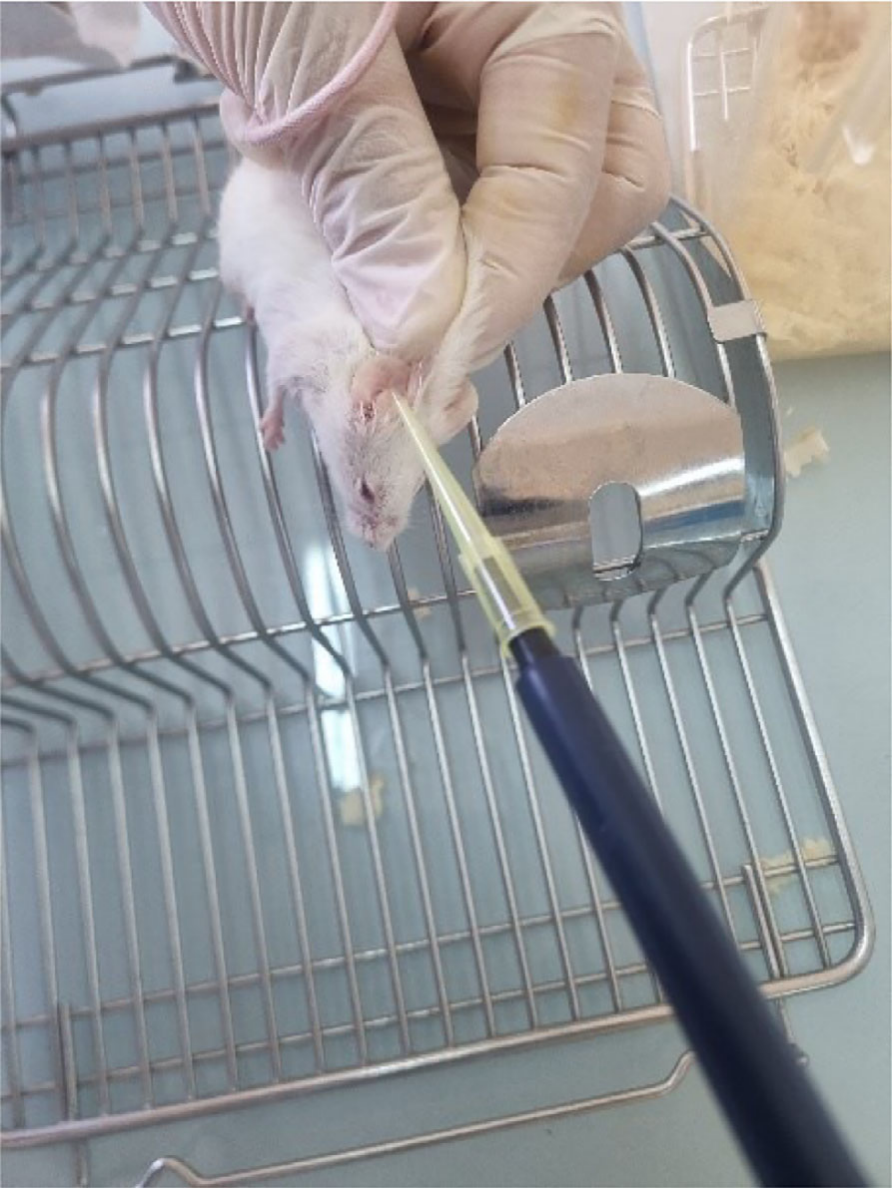
Ear edema application. All images are original and were created by the authors for this manuscript.

**FIGURE 14:**
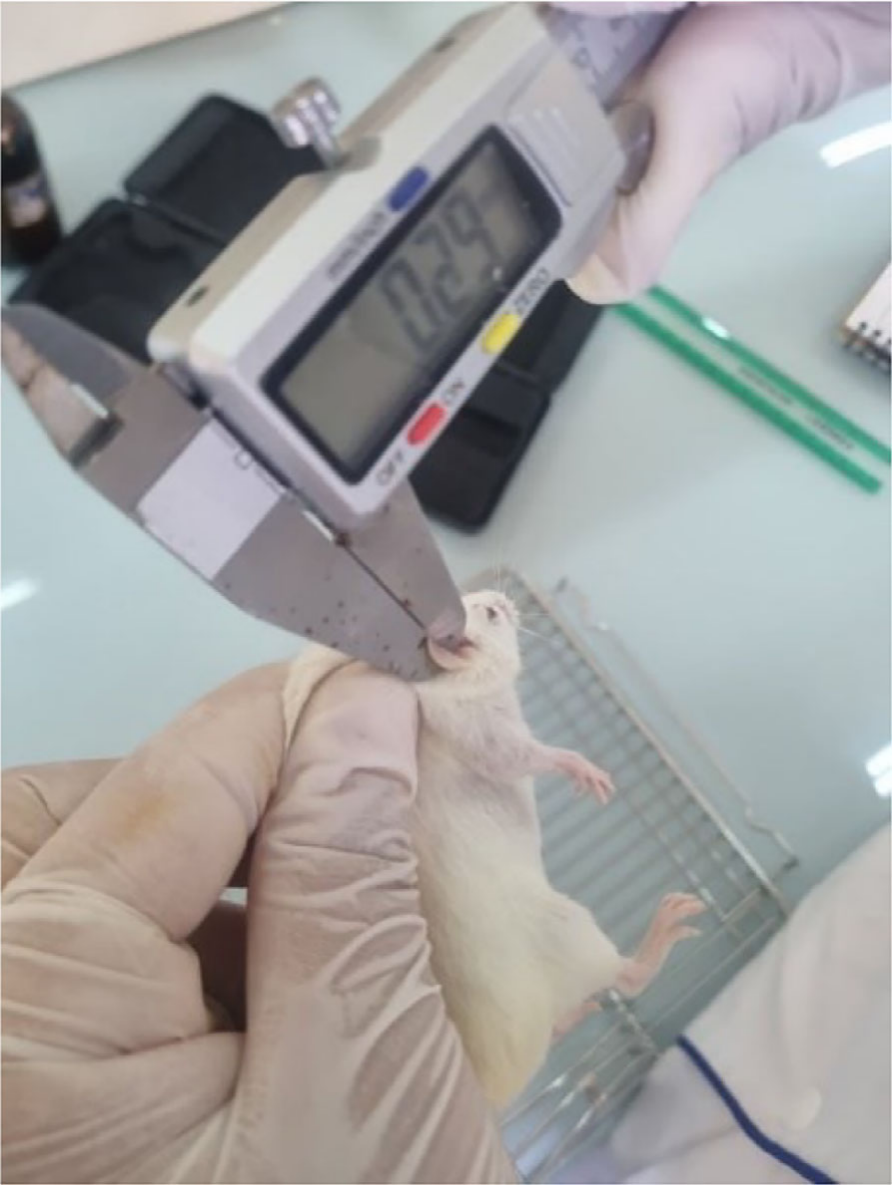
Measurement of the mouse's right ear with a digital caliper at 2 and 4 h post‐application. All images are original and were created by the authors for this manuscript.

Ear edema was induced by applying 20 µL of xylene to the inner surface of the mouse ear, whereas either Voltaren 1% cream or olive oil was simultaneously applied to the outer surface (Figure 13). The thickness of the right ear was measured with a digital caliper at two and 4 h post‐application (Figure 14). The anti‐inflammatory effect was expressed as the percentage reduction in edema. The percentage inhibition of edema, relative to the control group, was calculated using the following formula:

$$\begin{array}{ccc} & & \mathrm{Inflammation}\;\mathrm{Percentage}\left(\%\right)=\left[(\mathrm{Edema}\;\mathrm{in}\;\mathrm{control}\;\mathrm{group}/\right.\\ & & \;\left.-\mathrm{Edema}\;\mathrm{in}\;\mathrm{Treatment}\;\mathrm{Group}\;\mathrm{Edema}\;\mathrm{in}\;\mathrm{control}\;\mathrm{group})\times 100\right]. \end{array}$$

#### 4.8.2 Evaluation of the In Vivo Wound Healing Activity of Olive Oil

Wound healing is a complex process that occurs in three distinct yet sometimes overlapping phases:

- The first phase is marked by vascular and inflammatory responses.
- The second phase is tissue repairment, involving the regeneration of both dermis and epidermis tissues.
- The third phase encompasses the remodeling of the extracellular matrix and final maturation of the scar.

Wound healing can be broadly defined as the natural process by which damaged tissues regenerate, often leading to scar formation [47].

In this study, the effect of olive oil from the *O. europaea* L. species on wound healing was evaluated. Burns were induced on Wistar rats in the area between their shoulders, and a topical ointment formulated from this olive oil was applied for the pharmacological tests to assess its efficacy.

#### 4.8.3 Preparation of the Ointment

Ointments comprise homogeneous, semi‐solid preparations, formulated for topical application on the skin, either to provide localized effects or to promote the transdermal absorption of active ingredients [48]. In the current study, *O. europaea* olive oil was incorporated into a base mixture of vaseline and sodium benzoate to achieve a final concentration of 6% (Table 8). The proportions used in the preparation of the ointment are outlined in the table below:

**TABLE 8:** Composition of the ointment.

Component	Vaseline	Ointment 6%
Olive oil	0 g	3 g
Vaseline	49.925 g	46.925 g
Sodium benzoate	0.075 g	0.075 g
Total	50 g	50 g

*Note*: The ointments were packaged in airtight containers and stored at room temperature, away from light.

#### 4.8.4 Treatments

Biafine: This is a clinically approved cream that contains a topical emulsion with 0.67 g of trolamine per 100 g. Trolamine acts both as an analgesic and an emulsifier, and it is mainly indicated for the treatment of superficial first‐ and second‐degree burns. The cream was applied in a thick layer directly to the affected area to ensure full saturation of the skin. The 0.6% concentration of Biafine was selected because it is commonly used in clinical practice for superficial burns and is known to promote effective wound healing, as described in the literature [49]. To maintain consistency and comparability between the positive control and olive oil treatments, 20 µL of Biafine was applied.

Vaseline: A semi‐solid, white, translucent substance that is both tasteless and odorless, consisting of a blend of solid and liquid hydrocarbons. It is insoluble in water and alcohol but dissolves in non‐polar organic solvents. Vaseline is commonly used as a base in ointments for superficial applications and is not absorbed by the skin or mucous membranes [49]. The 20 µL dosage was chosen based on prior studies, such as Mayouf et al. [46], which demonstrated the effectiveness of this volume in producing significant anti‐inflammatory effects. The standard concentration for topical applications (1%) was used to ensure the treatment's comparability with other similar studies.

#### 4.8.5 Group Distribution

The rats were divided into nine groups, each comprising five individuals, to study the effects of the different treatments, as follows:

- Group 1 (T‐): Negative control group, comprising five rats without any treatment.
- Group 2 (T+): Positive control group, comprising five rats that received a topical application of 0.6% Biafine.
- Group 3 (T): This group received a topical application of Vaseline alone.
- Group 4: This group received an application of ointment containing 0.6% olive oil extracted from the Akerma variety at the green ripening stage.
- Group 5: This group received an application of ointment containing 0.6% olive oil extracted from the Akerma variety at the spotted ripening stage.
- Group 6: This group received an application of ointment containing 0.6% olive oil extracted from the Akerma variety at the black ripening stage.
- Group 7: This group was treated with an ointment containing 0.6% olive oil extracted from the Bouchouk Guergour variety at the green stage.
- Group 8: This group received an application of ointment containing 0.6% olive oil extracted from the Bouchouk Guergour variety at the spotted stage.
- Group 9: This group received a topical application of ointment containing 0.6% olive oil extracted from the Bouchouk Guergour variety at the black stage.

#### 4.8.6 Induction of Experimental Burns

**FIGURE 15:**
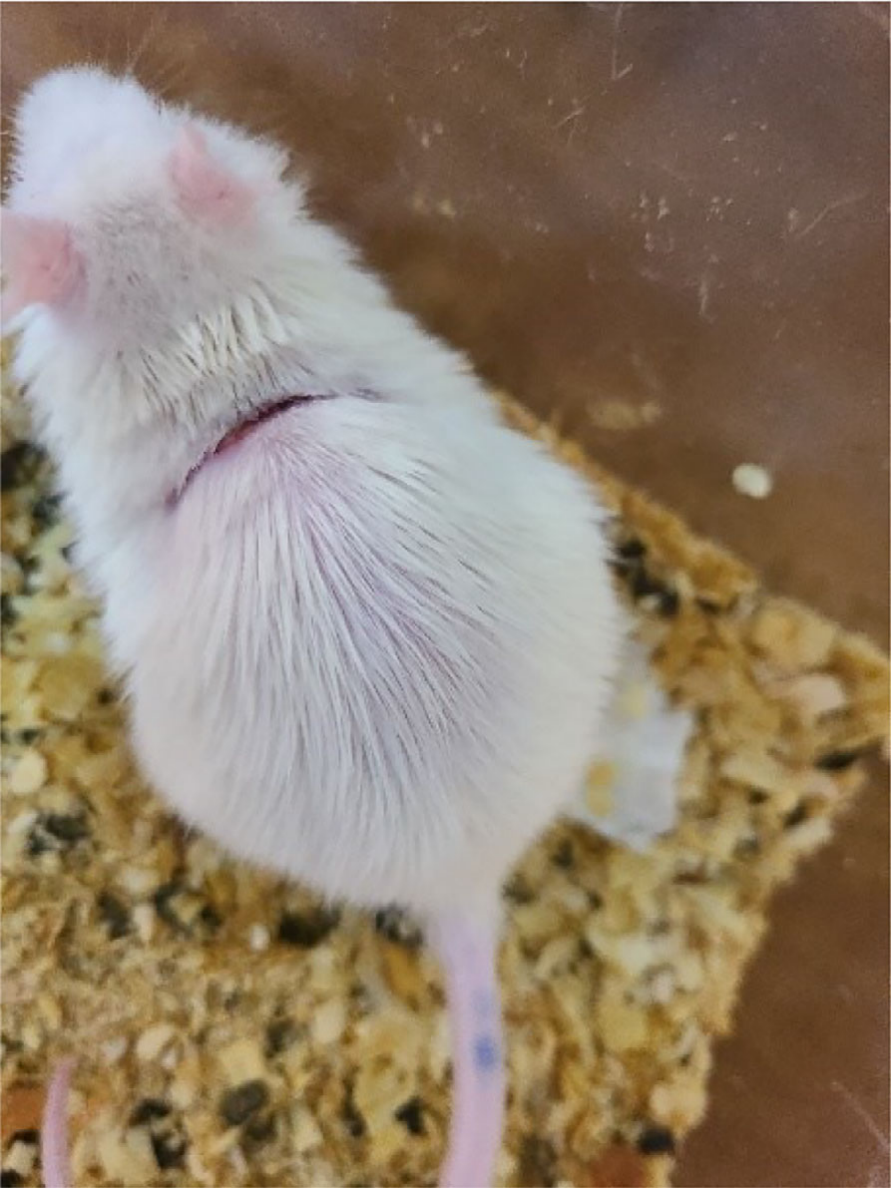
Induce burns on the shoulder of the rat. All images are original and were created by the authors for this manuscript.

The burns were induced following Hoşnuter et al. [50]. Before inducing the burn, the rats were locally anesthetized, and the area between the shoulders was shaved. A spatula with a 2 cm diameter, heated for 5 min using a Bunsen burner or a heating plate, was then applied to the shaved area without exerting any pressure for 15 s, in order to induce second‐degree burns (Figure 15).

#### 4.8.7 Evaluation of the Healing Process and Burns

The progression of burns was observed every 3 days to compare different healing processes and evaluate the surface area of each burn. The percentage of wound contraction was determined using the following equation: CC% = *J_0_* (*J_0_* − *J_n_*) × 100

- C%: Percentage of wound contraction
- *J_n_*: Size of the wound on Day *n*
- *J_0_*: Initial size of the wound on Day 1

The observation period lasted for 20 days, with a daily application of the ointment [51].

#### 4.8.8 Ethical Considerations

All animal experiments were conducted in accordance with international guidelines for the care and use of laboratory animals. The experimental protocols were approved by the Institutional Ethics Committee of the Faculty of Natural and Life Sciences, Ferhat Abbas University Sétif 1, Algeria (Approval No. CE‐FSNV‐2022‐017).

Male Wistar rats (180–220 g) and Swiss albino mice were obtained from the animal facility of the Faculty of Natural and Life Sciences. All efforts were made to minimize animal suffering and to reduce the number of animals used.

### 4.9 Statistical Analysis

The results of the tests were expressed as means and standard deviations. Significant differences for all parameters were set at *p* < 0.05 for the Analysis of Variance (ANOVA) test, followed by post‐hoc Tukey's HSD test, where applicable. The statistical analyses of data were performed in Statistica v8.0.

Statistical analysis was performed using one‐way analysis of variance (ANOVA) followed by Tukey's multiple comparison post hoc test. Data are expressed as mean ± standard deviation (SD). For the anti‐inflammatory assay, comparisons between treatments were performed at 2 and 4 h after xylene application. For the wound healing assay, statistical comparisons were carried out at each experimental time point. Differences were considered statistically significant at *p* < 0.05.

## Author Contributions

**Samir Sahli**: investigation, formal analysis, validation, writing – original draft. **Nabila Souilah**: writing – review and editing. **Hamdi Bendif**: writing – review and editing, funding and supervision. **Amar Benmahammed, Saliha Dahamna, Walid Elfalleh**: methodology and analysis. **Ramazan Erenler and İbrahim Demirtaş**: investigation and formal analysis. **Fehmi Boufahja**: review and editing. **Stefania Garzoli**: writing – review and editing and supervision. All authors have read and agreed to the published version of the manuscript.

## Ethics Statement

All protocols used in this study were approved by the Ethical Committee of Directorate General for Scientific Research and Technological Development at Algerian Ministry of Higher Education and Scientific Research (PRFU D00L05UN280120220001).

## Conflicts of Interest

The authors declare no conflicts of interest.

## Data Availability

The authors have nothing to report.
